# A Case Report on an Insurmountable Endodontic Problem: Internal Resorption

**DOI:** 10.7759/cureus.25126

**Published:** 2022-05-18

**Authors:** Swapnil Pawar, Prasad Patil, Tarun Kumar Singh, Swapnali Shinde, Mahesh Ghadage, Dipooja Patil

**Affiliations:** 1 Department of Endodontics, Yogita Dental College, Khed, IND; 2 Department of Endodontics, Al Ameen Dental College, Bijapur, IND; 3 Conservative Dentistry and Endodontics, All India Institute of Medical Sciences, Bathinda, IND; 4 Department of Preventive and Pediatric Dentistry, Child Dental Home, Mumbai, IND; 5 Department of Prosthodontics, Bharati Vidyapeeth Dental College and Hospital, Mumbai, IND; 6 Department of Conservative Dentistry and Endodontics, Bharati Vidyapeeth Dental College and Hospital, Mumbai, IND

**Keywords:** endodontics, regeneration, biodentine, cbct, internal resorption

## Abstract

Endodontists have a major problem when dealing with perforating internal resorption, which is an uncommon condition in permanent teeth. Success in treating a resorbed root can only be achieved if the root is properly diagnosed, removed, and treated. Cone-beam computed tomography was used to locate the resorptive lesion and assess its severity (CBCT). A maxillary canine with significant root perforation owing to internal resorption was successfully surgically treated in this case report.

## Introduction

Dentin, cementum, or bone may be lost due to either a physiologic or pathologic event, as described by the American Association of Endodontists' Glossary of Terms [1]. Most of the time, it is categorized as either internal (inflammatory) or external (replacement) (surface, inflammatory, and replacement) [2].

Dentinal tubules and tubules in the middle and apical thirds of the canal walls are progressively destroyed by odontoclastic activities in intraradicular internal resorption [3].

Clinically, there are no symptoms, and a diagnosis may be made through a normal X-ray. A 'pink tooth' may not be recognized until it has progressed to an advanced stage. The granulation tissue buildup in the coronal dentin is the cause of the pink tint, which compromises the crown [4]. A periodontal lesion may cause pain depending on the state of the pulp or the root's perforation. A radiolucent lesion inside the root canal space is detected radiographically as the cause of the irregular root canal outline, and its relationship to the root canal space does not shift after many radiographic pictures taken at various angulations [5].

Caries and periodontal infections, as well as iatrogenic operations such as improper restorations, vital pulp treatment with calcium hydroxide and vital root removals, and orthodontic techniques, have been theorized to be contributors to this condition [6]. Radiological techniques used to detect the presence of internal resorption have a significant rate of false-negative results. When it comes to the early identification of dental disease, “the non-invasive nature of cone-beam computed tomography (CBCT) and its high accuracy in detecting root lesions are two of the numerous benefits of using CBCT" [7].

A variety of products may be used to treat internal root resorption. These include metal-titanium alloys (MTAs), glass-ionomer cement, super EBA (a hydrophilic plastic polymer with barium salts), zinc oxide-eugenol-and-zinc acetate cement, and amalgam alloy.” It is possible, however, that the treatment biomaterial will impact the tooth's outcome [8]. For perforation involving surrounding periodontium, various regenerative materials can be used, such as allografts, alloplast, and xenografts, with the combination of collagen membrane to act as guided tissue regeneration. This paper conferrals perforating inflammatory internal resorption cases with a successful outcome and 12 months follow-up.

## Case presentation

**Figure 1:**
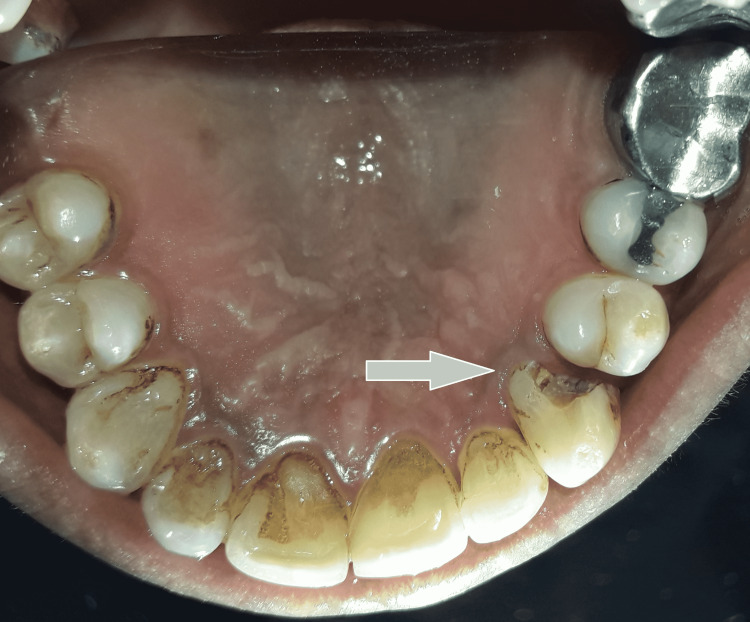
Preoperative intraoral clinic picture

A 38-year-old female patient came to the hospital with an excruciating ache in her upper left front teeth. The history of the patient's medical condition was not relevant. Clinically, there were disto-proximal caries in 23 and mesio-proximal caries in 24, as well as mild tenderness on percussion in 23, as well as a grade I mobility, and a 5mm periodontal pocket in 22 and 23 (Figure 1).

**Figure 2:**
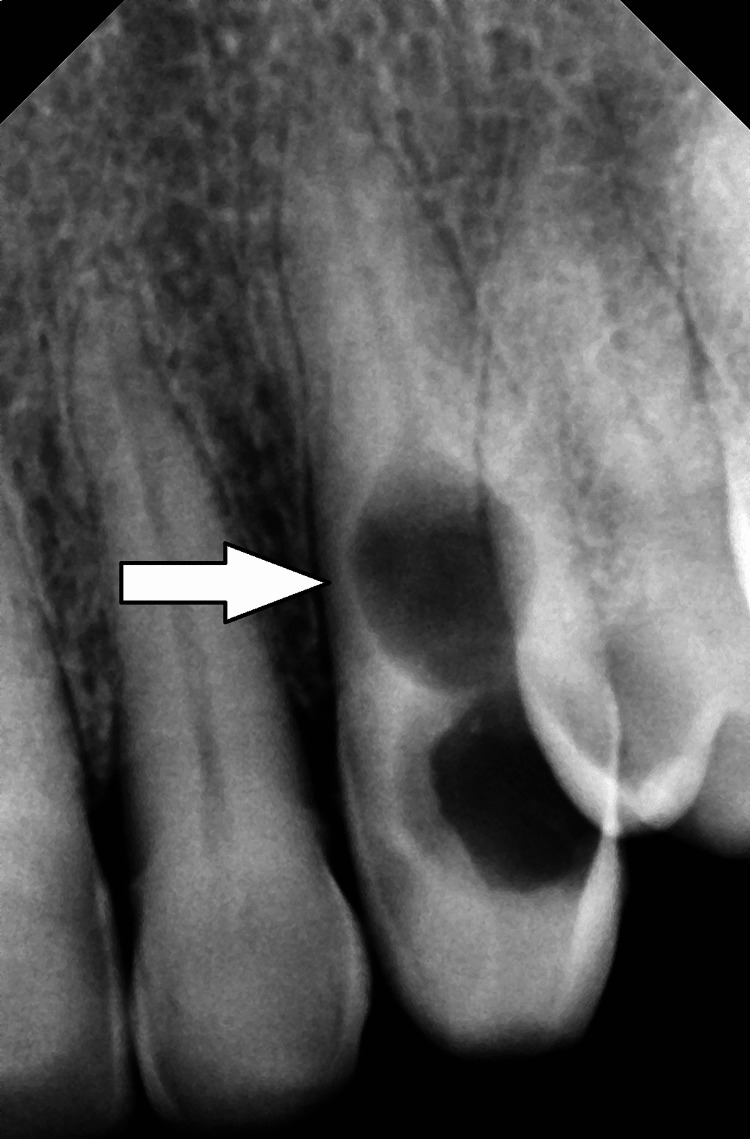
Preoperative intraoral periapical radiograph

The sinus tracts were not found in that area. Figure 2 shows an area of radiolucency in the root canal's cervical third that could not be traced through the lesion on radiographs. 

**Figure 3:**
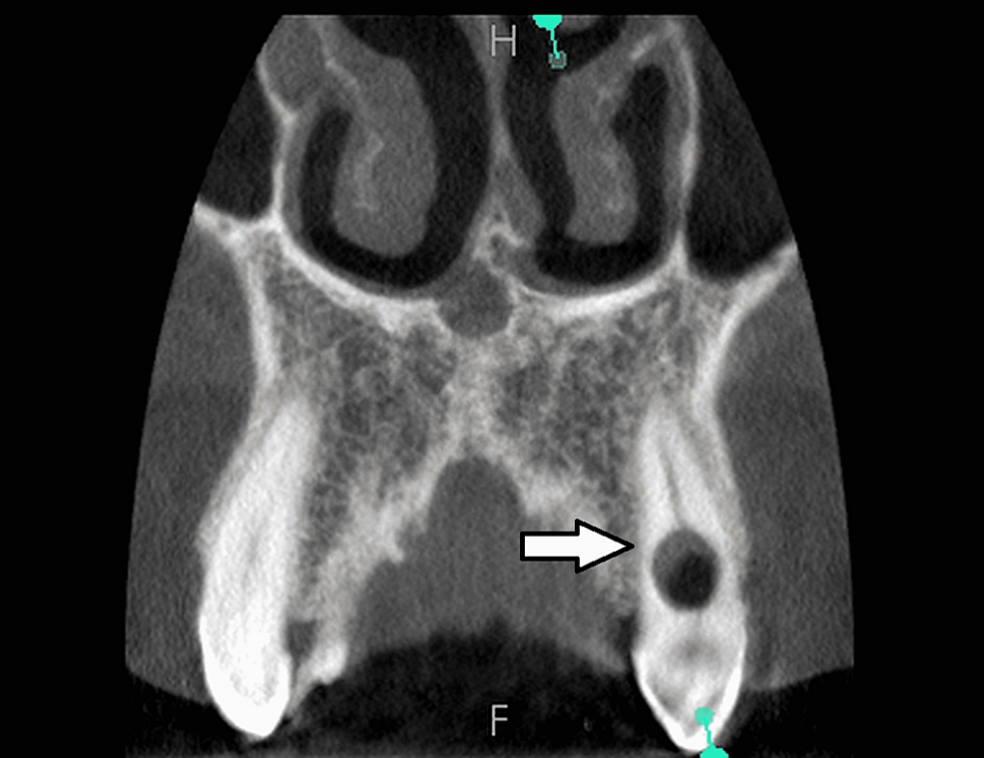
Cone-beam computed tomographic scan of the resorption site

The diagnosis of internal resportion was made based on the patient's history and the lesion's radiological findings. After obtaining the patient's permission, a CBCT scan was recommended to determine the location, type, and severity of the lesion. A mixed intra-bony defect was formed within 23 and 22 (Figure 3). The diagnosis served as the foundation for developing a therapy strategy. Periodontal surgery was performed after the endodontic treatment.

**Figure 4:**
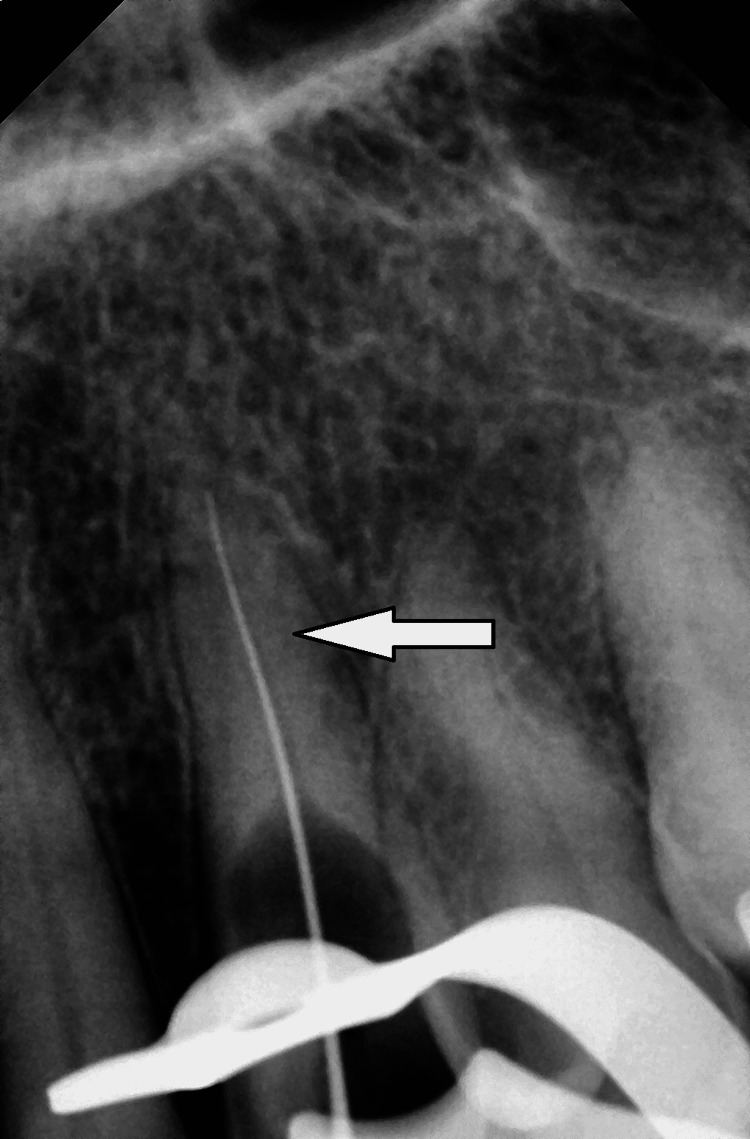
Working length determination

Root planing and scaling were both completed to achieve a smooth surface. Two percent lignocaine with 1:80000 adrenaline was injected, and tooth 23 was isolated under a rubber dam. Using a round bur, we removed caries and then prepared the access cavity. Radiological confirmation of radiographic working length was made using a 10 k file (Mani Inc., Japan) from the Root ZX apex locator (Figure 4).

**Figure 5:**
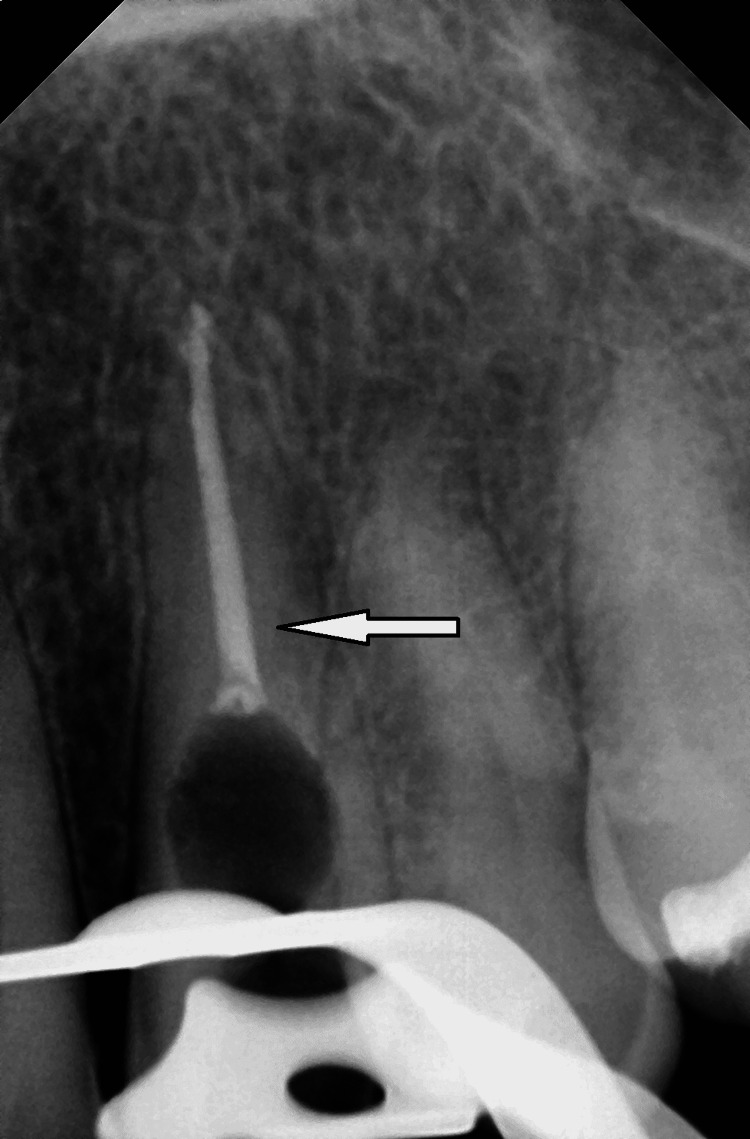
Obturation radiograph

Protaper Next (Dentsply Maillefer, Switzerland) rotary files up to X4 were used for root canal preparation. After using 3% sodium hypochlorite (Prime Dental Products, India) to clean the canal and resorption lacunae, an irritant was activated with Endoactivator (Dentsply Maillefer, Switzerland). Calcium hydroxide dressing and sterile paper points were used to sterilize it, according to the instructions on the packaging. Cavit gave a short-term fix (3M ESPE St. Paul, MN, USA). Calcium hydroxide was withdrawn, the access cavity reopened, and the last irrigation was performed on the patient after two consecutive appointments of calcium hydroxide. Mastercone was selected and confirmed radiographically. Ah plus sealer (Dentsply, Konstanz, Germany) was mixed properly and applied using lentulo-spiral (size 30 Mani Inc., Japan). A down pack of gutta percha was used to obturate the resorption cavity using an E & Q pen (Meta Biomed Co., Seoul, Korea) (Figure 5).

**Figure 6:**
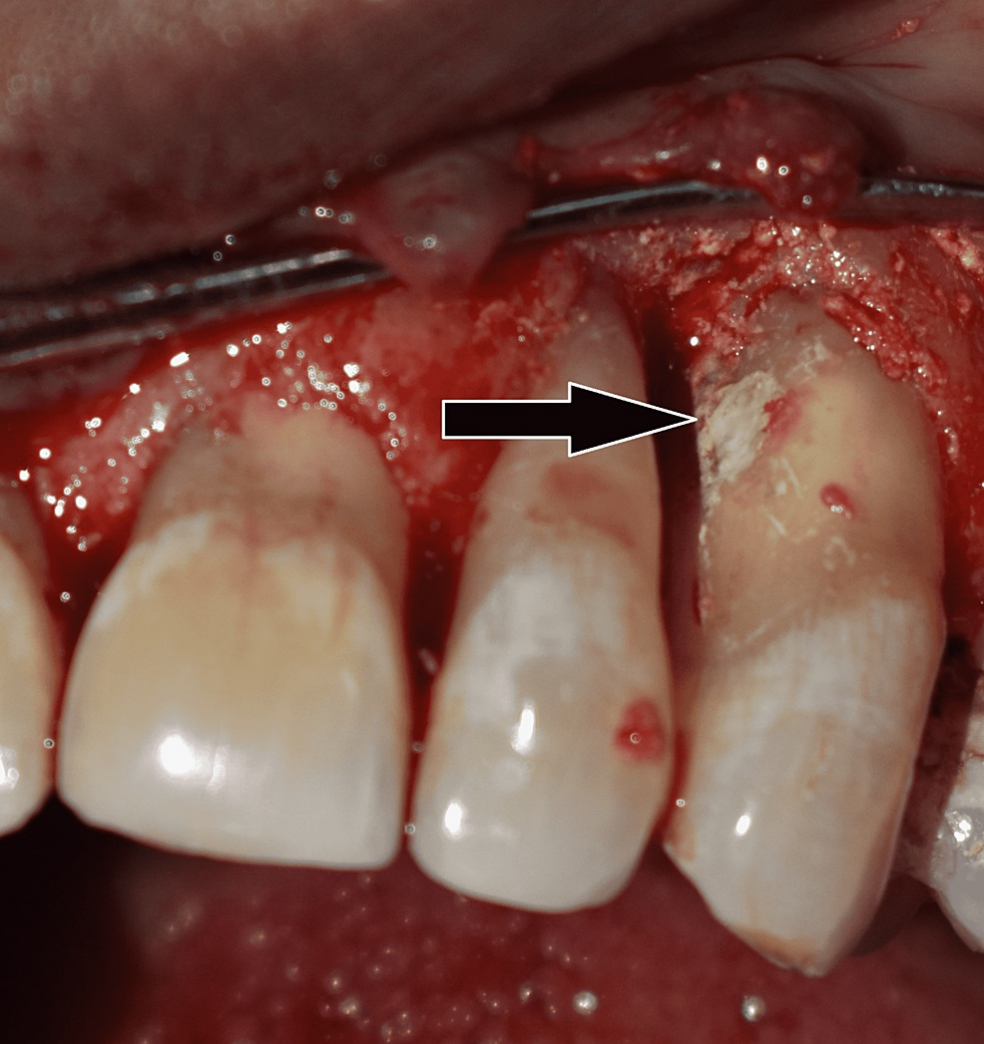
Lesion sealed with biodentine

When the patient returned to the dentist's office after 15 days, a crevicular incision was made from the right central incisor to the left second premolar, and a full-thickness mucoperiosteal flap was raised under local anaesthesia. Gracey curettes were used to degranulate and debride the wound completely. The intrabony defect was irrigated with saline, and perforation in 23 was fully accessed. To strengthen the tooth structure, Biodentine (Septodont Saint Maur-des Fosses, France) was used to fill the resorption access cavity with an MTA carrier, then condensed against the resorption wall using a root canal spreader (Figure 6).

**Figure 7:**
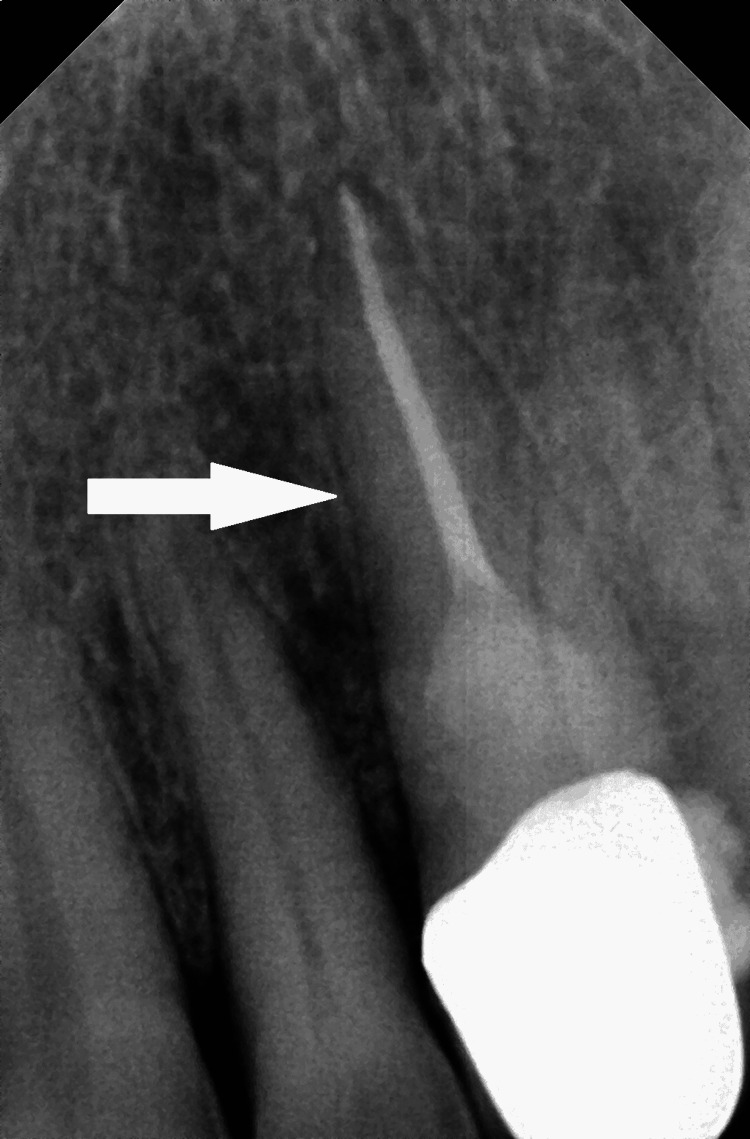
12 months follow up radiograph

Caries was removed and restored with resin-modified GIC with 24. Pre-suturing was done, DFDBA (Demineralized Freeze-Dried Bone Allograft) with L-PRF (leukocyte- and platelet-rich fibrin) combination were placed in 22 and 23 regions, as there were combined 2 walls intra-bony defect and PRF membrane was placed over it to act as a GTR (guided tissue regeneration). The flap was repositioned, and interrupted sutures were used. COE pack was placed. The patient was recalled after seven days, and after satisfactory wound healing was seen, sutures were removed. The tooth was restored using resin composite Filtex Z 250 XT (3M ESPE, St. Paul, MN, USA). After six months all-ceramic crown was cemented with 23. A 12-month follow-up shows complete healing on a radiograph (Figure 7).

## Discussion

Patients with severe and perforating root resorption are particularly difficult to treat because of the complexity of their condition. 3D CBCT has been demonstrated to help identify the difficulty of therapy, as well as provide an accurate prognosis based on the amount and severity of the lesion [9].

It is best to proceed with conventional root canal treatments as soon as the diagnosis and prognosis have been confirmed [10]. An important part of the root canal therapy process is to inhibit any further cell activity. Furthermore, calcium hydroxide and sodium hypochlorite must work together to remove organic material from the root canals [11].

Agitating irrigation solutions using an endocativator facilitates thorough penetration of the root canal system due to its intricate anatomical design [12]. Because the resorptive site's tooth structure is thin and fragile, bioactive material, such as Biodentine, has been employed to support it. It can deposit apatite like calcium phosphate crystals on the surface. This results in an improved interface between biodentine, dentin substitute, and phosphate-rich hard tooth structure. It can be easily manipulated, has better consistency, safety handling with a favourable setting time of about 12 min, and high push-out strength than MTA [13].

DFDBA (Demineralized Freeze-Dried Bone Allograft) is the most widely used and studied bone allograft with properties of osteoconduction and osteoinduction. Osteoconduction is where bone graft particles act as a scaffold to host bone cells. By causing undifferentiated mesenchymal cells in the bone matrix to undergo osteoblast differentiation, BMPs 2, 4, and 7 have the osteoinductive ability. Bone morphogenetic proteins (BMPs) are deposited and trapped inside the bone matrix when it develops and remineralizes. Improved handling and stability of graft by using PRF and DFDBA (Demineralized Freeze-Dried Bone Allograft) together [14]. Fibrin meshwork made up of cytokines, glycosaminoglycans, and growth factors like platelet-derived growth factor (PDGF) is called platelet-rich fibrin (PRF) (PDGF).

Mitosis-inducing factors like PDGF may be used alone or in conjunction with a progression, factor to stimulate osteoblast proliferation. Neo-angiogenesis is aided by the soluble angiogenesis factors vascular endothelial growth factor (VEGF), angiopoietin, and platelet-derived growth factor (PDGF), all of which are contained in fibrin gel. The fibrin structure of PRF may be to blame for the slow release of growth factors, including PDGF, transforming growth factor (TGF), and matrix glycoproteins. It is possible to utilize PRF alone or in conjunction with other bone grafts to take advantage of its additional benefits [15].

In the present case report, the internal resorption cavity with perforation was repaired using Biodentine in the maxillary canine, and the bone defect was filled with DFDBA along with PRF and PRF membrane. 

## Conclusions

For a good treatment result, early identification, removal of the source, and effective treatment of the resorbed root are critical. Dentinal resorbed teeth may be completely rehabilitated thanks to the latest advancements in endodontic diagnostics, irrigation activation, obturating materials, and the use of bioactive materials.
